# Crystal structure of the unusual coordination polymer *catena*-poly[[gold(I)-μ-1,2-bis­(di­phenyl­phosphino­thio­yl)ethane-κ^2^ *S*:*S*′] di­bromido­aurate(I)]^1^

**DOI:** 10.1107/S2056989020013675

**Published:** 2020-10-20

**Authors:** Christina Taouss, Marina Calvo, Peter G. Jones

**Affiliations:** a Institut für Anorganische und Analytische Chemie, Technische Universität Braunschweig, Hagenring 30, D-38106 Braunschweig, Germany

**Keywords:** crystal structure, polymer, phosphine sulfide, gold

## Abstract

In the title compound, {[Au(C_26_H_24_P_2_S_2_)][AuBr_2_]}_*n*_, the gold(I) centres of the cation are coordinated by the P=S groups of the di­sulfide ligands to form a chain polymer parallel to the *c* axis. Both independent gold atoms lie on the same twofold axis, and the midpoint of the H_2_C—CH_2_ bond lies on an inversion centre. The anions flank the polymeric chain; they are connected to it by short aurophilic inter­actions and C—H⋯Br contacts, and to each other by Br⋯Br contacts.

## Abstract 2

In the title compound, the gold(I) centres of the cation are coordinated by the P=S groups of the di­sulfide ligands to form a chain polymer parallel to the *c* axis. Both independent gold atoms lie on the same twofold axis, and the midpoint of the H_2_C—CH_2_ bond lies on an inversion centre. The anions flank the polymeric chain; they are connected to it by short aurophilic inter­actions and C—H⋯Br contacts, and to each other by Br⋯Br contacts.

## Chemical context   

Although phosphane sulfides are known to act as ligands towards gold(I) centres, not many complexes have been structurally characterized in which two such ligands coordinate to gold(I). A search of the Cambridge Database (2019 Version, *ConQuest* 2.0.5) revealed only three structures involving the cation [(Ph_3_P=S)_2_Au]^+^; the PO_2_F_2_^−^ salt (LeBlank *et al.*, 1997 ▸), the nitrate (Jones & Geissler, 2016*a* ▸) and a bis­(methyl­sulfon­yl)amide salt (Jones & Geissler, 2016*b* ▸). Cationic 1:1 complexes of gold(I) with diphosphane di­sulfides can only be achieved if the ligand geometry allows for linear coordination at the gold atom, which is not generally the case unless suitable spacers, such as ferrocene units or other metal centres, are present (Gimeno *et al.*, 2000 ▸, and references therein; Parkanyi & Besenyei, 2017 ▸; Wang & Fackler, 1990 ▸).
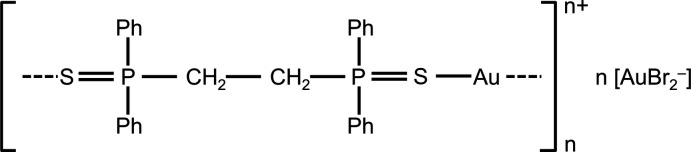


In the course of our studies of phosphane chalcogenide complexes of gold (Upmann *et al.*, 2019 ▸, and references therein) we planned to study complexes of the diphosphane di­sulfides 1,2-bis­(di­phenyl­phosphino­thio­yl)ethane [previously known as 1,2-bis­(di­phenyl­phosphino)ethane di­sulfide; dppeS_2_] and bis­(di­phenyl­thio­phosphino­yl)methane [prev­iously known as bis­(di­phenyl­phosphino)methane di­sulfide; dppmS_2_] with gold(I) halide fragments AuBr and AuCl, with particular attention to the mononuclear complexes. This succeeded to some extent; we were able to isolate and determine the structure of dppmS_2_AuCl, the isotypic dppmS_2_AuBr and its oxidation product with bromine [(dppmS_2_)AuBr_2_]^+^ [AuBr_4_]^−^ (Jones *et al.*, 2020*a* ▸,*b* ▸,*c* ▸, respectively), but yields were poor and it was clear that scrambling reactions were a problem. With dppeS_2_ even less was achieved, but a few thin needles, isolated from the attempted synthesis of dppeS_2_AuBr, proved to be an unusual coordination polymer [(dppeS_2_)Au]_*n*_^*n*+^·*n*[AuBr_2_]^−^, the structure of which we report here.

## Structural commentary   

**Figure 1:**
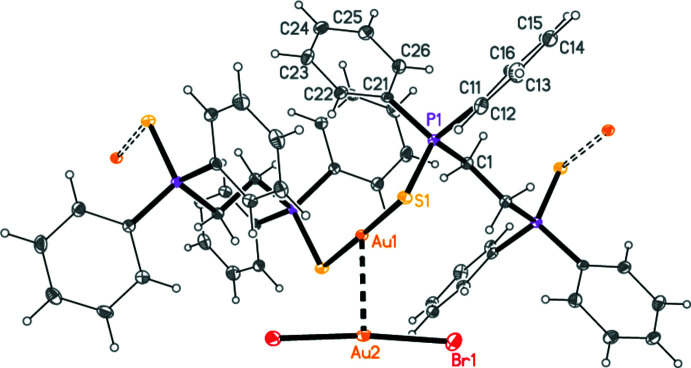
Structure of the title complex in the crystal; the asymmetric unit is numbered. The aurophilic contact and the S—Au connections to the next gold atoms in the polymer are indicated by filled and open dashed bonds, respectively.

The title compound is shown in Fig. 1 ▸. The cation has the stoichiometry [dppeS_2_Au]^+^, and forms a chain polymer (⋯Au—S=PCH_2_CH_2_P=S⋯)_*n*_ parallel to the *c* axis; the anion is [AuBr_2_]^−^. Both gold atoms lie on twofold axes 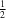, *y*, 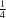 and show the linear coordination geometry expected for Au^I^; the midpoint of the central H_2_C—CH_2_ bond lies on the inversion centre 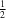, 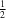, 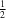. Bond lengths and angles may be considered normal; for a selection, see Table 1 ▸. Coordination polymers are scarce for diphosphane di­sulfide ligands (see below).

## Supra­molecular features   

**Figure 2:**
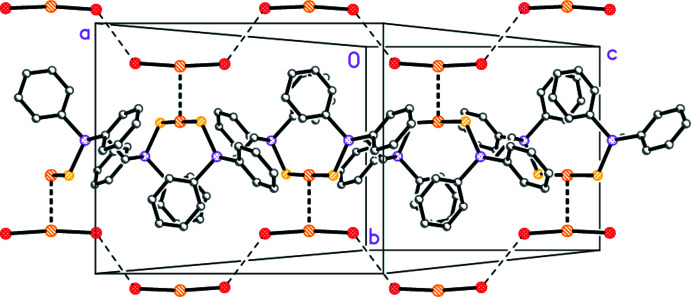
The polymeric structure of the title compound, viewed perpendicular to the (101) plane. Aurophilic contacts are shown as thick bonds and Br⋯Br contacts as thin dashed bonds. Hydrogen atoms are omitted for clarity.

The gold atoms of the cation and anion are connected *via* a short aurophilic contact of 2.9622 (3) Å, and the anions thus flank the cation polymer (Fig. 2 ▸). Neighbouring anions are connected by short Br⋯Br contacts of 3.7424 (8) Å (operator 1 − *x*, 2 − *y*, 1 − *z*), and also provide links to adjacent polymers (not shown in Fig. 2 ▸). We have previously noted an example of short contacts between di­bromo­aurate(I) anions (Döring & Jones, 2013 ▸); for a further example, see Beno *et al.* (1990 ▸). We have also described Br⋯Br and Cl⋯Cl contacts in a series of tetra­bromido­aurate(III) and tetra­chlorido­aurate(III) salts (Döring & Jones, 2016 ▸).

Two C—H⋯Br contacts between cation and anions are sufficiently short and linear to be considered ‘weak’ hydrogen bonds (Table 2 ▸), and thus to contribute further cohesion to the structure, but are omitted from Fig. 2 ▸ for clarity.

## Database survey   

A database search (CSD 2019 Version, *ConQuest* 2.0.5) found 11 hits for systems involving two P=S units bonded to Au^I^. The P=S bond lengths range from 1.985–2.039, av. 2.018 Å, and the S—Au bond lengths from 2.275–2.317, av. 2.296 Å. The only other coordination polymer found for a diphosphane di­sulfide was [(CuCN)_2_(dppeS_2_)]_*n*_ (Zhou *et al.*, 2006 ▸), a two-dimensional polymer involving four-coordinate Cu centres.

## Synthesis and crystallization   

The compound arose from an attempt to synthesize dppeS_2_AuBr. A solution of thtAuBr (tht = tetra­hydro­thio­phene; 0.775 g, 2.12 mmol) in CH_2_Cl_2_ (50 ml) was added to dppeS_2_ (0.981 g, 2.12 mmol) dissolved in CH_2_Cl_2_ (50 ml). After stirring for 1 h, the solvent was removed, and the solid thus obtained was dried under vacuum and recrystallized from di­chloro­methane/*n*-pentane. The elemental analysis was approximately correct for the expected stoichiometry: calculated, C 42.23%, H 3.27%, S 8.67%; found, C 43.22%, H 3.87%, S 8.19%. However, attempts to obtain crystals suitable for X-ray structure analysis (by evaporation from a solution in CH_2_Cl_2_) led only to a few very thin needles of the title compound, with overall stoichiometry dppeS_2_(AuBr)_2_.

## Refinement   

Crystal data, data collection and structure refinement details are summarized in Table 3 ▸. Hydrogen atoms were included using a riding model starting from calculated positions (C—H_aromatic_ = 0.95, C—H_methyl­ene_ = 0.99 Å). The *U*_iso_(H) values were fixed at 1.2 times the equivalent *U*_iso_ value of the parent carbon atoms.

## Supplementary Material

Crystal structure: contains datablock(s) I, global. DOI: 10.1107/S2056989020013675/ex2037sup1.cif

Structure factors: contains datablock(s) I. DOI: 10.1107/S2056989020013675/ex2037Isup2.hkl

CCDC reference: 2036798

Additional supporting information:  crystallographic information; 3D view; checkCIF report

## Figures and Tables

**Table 1:** Selected geometric parameters (Å, °)

Au1—S1	2.3125 (9)	Br1⋯Br1^i^	3.7424 (8)
Au1⋯Au2	2.9622 (3)	P1—S1	2.0097 (12)
Au2—Br1	2.3746 (5)	C1—C1^ii^	1.528 (6)
			
S1—Au1—S1^iii^	178.43 (4)	Br1—Au2⋯Au1	93.201 (11)
S1—Au1⋯Au2	90.78 (2)	Au2—Br1⋯Br1^i^	126.21 (2)
Br1—Au2—Br1^iii^	173.60 (2)	P1—S1—Au1	98.34 (4)

Symmetry codes: (i) 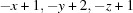; (ii) 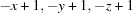; (iii) 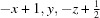.

**Table 2:** Hydrogen-bond geometry (Å, °)

*D*—H⋯*A*	*D*—H	H⋯*A*	*D*⋯*A*	*D*—H⋯*A*
C16—H16⋯Br1^ii^	0.95	2.81	3.712 (4)	159
C26—H26⋯Br1^ii^	0.95	2.89	3.775 (4)	155

Symmetry code: (ii) 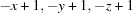.

**Table 3:** Experimental details

Crystal data	
Chemical formula	[Au(C_26_H_24_P_2_S_2_)][AuBr_2_]
*M*_r_	1016.26
Crystal system, space group	Monoclinic, *C*2/*c*
Temperature (K)	100
*a*, *b*, *c* (Å)	21.4112 (8), 11.9708 (2), 13.7726 (4)
β (°)	128.316 (7)
*V* (Å^3^)	2769.7 (3)
*Z*	4
Radiation type	Cu *K*α
μ (mm^−1^)	25.63
Crystal size (mm)	0.12 × 0.005 × 0.005

Data collection	
Diffractometer	Oxford Diffraction Xcalibur, Atlas, Nova
Absorption correction	Multi-scan (*CrysAlis PRO*; Oxford Diffraction, 2010 ▸)
*T*_min_, *T*_max_	0.387, 1.000
No. of measured, independent and observed [*I* > 2σ(*I*)] reflections	27632, 2873, 2630
*R*_int_	0.037
(sin θ/λ)_max_ (Å^−1^)	0.629

Refinement	
*R*[*F* ^2^ > 2σ(*F* ^2^)], *wR*(*F* ^2^), *S*	0.021, 0.053, 1.06
No. of reflections	2873
No. of parameters	155
H-atom treatment	H-atom parameters constrained
Δρ_max_, Δρ_min_ (e Å^−3^)	1.42, −0.99

Computer programs: *CrysAlis PRO* (Oxford Diffraction, 2010 ▸), *SHELXS97* (Sheldrick, 2008 ▸), *SHELXL2018/3* (Sheldrick, 2015 ▸) and *XP* (Siemens, 1994 ▸).

## supplementary crystallographic information

### Crystal data

**Table:**

[Au(C_26_H_24_P_2_S_2_)][AuBr_2_]	*F*(000) = 1880
*M**_r_* = 1016.26	*D*_x_ = 2.437 Mg m^−^^3^
Monoclinic, *C*2/*c*	Cu *K*α radiation, λ = 1.54184 Å
*a* = 21.4112 (8) Å	Cell parameters from 17272 reflections
*b* = 11.9708 (2) Å	θ = 4.1–75.8°
*c* = 13.7726 (4) Å	µ = 25.63 mm^−^^1^
β = 128.316 (7)°	*T* = 100 K
*V* = 2769.7 (3) Å^3^	Needle, colourless
*Z* = 4	0.12 × 0.01 × 0.01 mm

### Data collection

**Table:**

Oxford Diffraction Xcalibur, Atlas, Nova diffractometer	2873 independent reflections
Radiation source: Nova (Cu) X-ray Source	2630 reflections with *I* > 2σ(*I*)
Mirror monochromator	*R*_int_ = 0.037
Detector resolution: 10.3543 pixels mm^-1^	θ_max_ = 76.0°, θ_min_ = 4.5°
ω scans	*h* = −26→26
Absorption correction: multi-scan (CrysAlisPro; Oxford Diffraction, 2010)	*k* = −14→15
*T*_min_ = 0.387, *T*_max_ = 1.000	*l* = −17→16
27632 measured reflections	

### Refinement

**Table:**

Refinement on *F*^2^	Primary atom site location: structure-invariant direct methods
Least-squares matrix: full	Secondary atom site location: difference Fourier map
*R*[*F*^2^ > 2σ(*F*^2^)] = 0.021	Hydrogen site location: inferred from neighbouring sites
*wR*(*F*^2^) = 0.053	H-atom parameters constrained
*S* = 1.06	*w* = 1/[σ^2^(*F*_o_^2^) + (0.0283*P*)^2^ + 12.9976*P*] where *P* = (*F*_o_^2^ + 2*F*_c_^2^)/3
2873 reflections	(Δ/σ)_max_ = 0.001
155 parameters	Δρ_max_ = 1.42 e Å^−^^3^
0 restraints	Δρ_min_ = −0.99 e Å^−^^3^

### Special details

**Table:**

Geometry. All esds (except the esd in the dihedral angle between two l.s. planes) are estimated using the full covariance matrix. The cell esds are taken into account individually in the estimation of esds in distances, angles and torsion angles; correlations between esds in cell parameters are only used when they are defined by crystal symmetry. An approximate (isotropic) treatment of cell esds is used for estimating esds involving l.s. planes.

### Fractional atomic coordinates and isotropic or equivalent isotropic displacement parameters (Å^2^)

**Table:**

	*x*	*y*	*z*	*U*_iso_*/*U*_eq_	
Au1	0.500000	0.61954 (2)	0.250000	0.01687 (7)	
Au2	0.500000	0.86699 (2)	0.250000	0.01950 (7)	
Br1	0.47685 (3)	0.87807 (3)	0.39751 (4)	0.03124 (11)	
P1	0.60133 (5)	0.46450 (7)	0.49985 (8)	0.01384 (16)	
S1	0.61453 (5)	0.61690 (7)	0.45351 (8)	0.01915 (17)	
C1	0.50616 (19)	0.4520 (3)	0.4701 (3)	0.0162 (7)	
H1A	0.462644	0.452353	0.379701	0.019*	
H1B	0.504140	0.380043	0.503474	0.019*	
C11	0.6769 (2)	0.4432 (3)	0.6634 (3)	0.0167 (7)	
C12	0.7460 (2)	0.5083 (3)	0.7329 (4)	0.0214 (8)	
H12	0.752518	0.568996	0.695455	0.026*	
C13	0.8047 (2)	0.4839 (4)	0.8566 (4)	0.0290 (9)	
H13	0.851649	0.528125	0.903677	0.035*	
C14	0.7962 (2)	0.3962 (4)	0.9128 (4)	0.0267 (8)	
H14	0.836922	0.380547	0.997887	0.032*	
C15	0.7280 (2)	0.3310 (3)	0.8446 (3)	0.0227 (7)	
H15	0.721865	0.270674	0.882930	0.027*	
C16	0.6684 (2)	0.3542 (3)	0.7197 (3)	0.0198 (7)	
H16	0.621780	0.309224	0.672798	0.024*	
C21	0.61000 (19)	0.3529 (3)	0.4211 (3)	0.0154 (6)	
C22	0.6509 (2)	0.3700 (3)	0.3737 (4)	0.0220 (7)	
H22	0.670919	0.441911	0.377148	0.026*	
C23	0.6622 (3)	0.2805 (4)	0.3212 (4)	0.0287 (8)	
H23	0.689530	0.291899	0.287999	0.034*	
C24	0.6338 (2)	0.1754 (3)	0.3171 (4)	0.0274 (8)	
H24	0.641465	0.114817	0.280842	0.033*	
C25	0.5943 (2)	0.1586 (3)	0.3657 (4)	0.0264 (8)	
H25	0.575405	0.086098	0.363559	0.032*	
C26	0.5819 (3)	0.2461 (3)	0.4176 (4)	0.0237 (8)	
H26	0.554451	0.233937	0.450538	0.028*	

### Atomic displacement parameters (Å^2^)

**Table:**

	*U*^11^	*U*^22^	*U*^33^	*U*^12^	*U*^13^	*U*^23^
Au1	0.01921 (11)	0.01291 (10)	0.01950 (11)	0.000	0.01250 (9)	0.000
Au2	0.01812 (11)	0.01358 (10)	0.02586 (12)	0.000	0.01316 (9)	0.000
Br1	0.0371 (2)	0.0274 (2)	0.0408 (3)	−0.00694 (16)	0.0299 (2)	−0.00832 (17)
P1	0.0127 (4)	0.0131 (4)	0.0155 (4)	−0.0004 (3)	0.0086 (3)	−0.0009 (3)
S1	0.0192 (4)	0.0151 (4)	0.0211 (4)	−0.0024 (3)	0.0115 (4)	−0.0003 (3)
C1	0.0129 (15)	0.0177 (16)	0.0170 (16)	−0.0012 (12)	0.0088 (13)	−0.0015 (13)
C11	0.0153 (15)	0.0178 (16)	0.0192 (17)	0.0005 (12)	0.0117 (14)	−0.0041 (13)
C12	0.0180 (17)	0.0258 (19)	0.0236 (19)	−0.0060 (14)	0.0144 (16)	−0.0051 (15)
C13	0.0168 (17)	0.041 (2)	0.022 (2)	−0.0063 (16)	0.0087 (16)	−0.0067 (17)
C14	0.0164 (17)	0.040 (2)	0.0161 (18)	0.0064 (15)	0.0063 (15)	−0.0014 (16)
C15	0.0236 (18)	0.0245 (18)	0.0183 (18)	0.0060 (14)	0.0121 (15)	0.0027 (14)
C16	0.0170 (16)	0.0182 (16)	0.0197 (18)	0.0009 (13)	0.0091 (14)	−0.0008 (13)
C21	0.0139 (15)	0.0147 (15)	0.0160 (16)	0.0032 (12)	0.0085 (14)	0.0022 (12)
C22	0.0235 (18)	0.0204 (17)	0.0269 (19)	0.0039 (13)	0.0181 (16)	0.0025 (14)
C23	0.033 (2)	0.032 (2)	0.035 (2)	0.0043 (17)	0.0276 (19)	0.0005 (18)
C24	0.032 (2)	0.0236 (19)	0.026 (2)	0.0078 (16)	0.0173 (17)	−0.0010 (15)
C25	0.034 (2)	0.0164 (17)	0.028 (2)	−0.0033 (15)	0.0186 (18)	−0.0053 (15)
C26	0.028 (2)	0.0203 (19)	0.026 (2)	−0.0037 (13)	0.0187 (18)	−0.0027 (14)

### Geometric parameters (Å, º)

**Table:**

Au1—S1	2.3125 (9)	C13—H13	0.9500
Au1—S1^i^	2.3125 (9)	C14—C15	1.387 (6)
Au1—Au2	2.9622 (3)	C14—H14	0.9500
Au2—Br1	2.3746 (5)	C15—C16	1.393 (5)
Au2—Br1^i^	2.3746 (5)	C15—H15	0.9500
Br1—Br1^ii^	3.7424 (8)	C16—H16	0.9500
P1—C11	1.800 (4)	C21—C22	1.394 (5)
P1—C21	1.801 (4)	C21—C26	1.401 (5)
P1—C1	1.819 (3)	C22—C23	1.394 (5)
P1—S1	2.0097 (12)	C22—H22	0.9500
C1—C1^iii^	1.528 (6)	C23—C24	1.384 (6)
C1—H1A	0.9900	C23—H23	0.9500
C1—H1B	0.9900	C24—C25	1.381 (6)
C11—C16	1.395 (5)	C24—H24	0.9500
C11—C12	1.399 (5)	C25—C26	1.384 (5)
C12—C13	1.382 (6)	C25—H25	0.9500
C12—H12	0.9500	C26—H26	0.9500
C13—C14	1.383 (6)		
			
S1—Au1—S1^i^	178.43 (4)	C14—C13—H13	119.5
S1—Au1—Au2	90.78 (2)	C13—C14—C15	119.9 (4)
S1^i^—Au1—Au2	90.78 (2)	C13—C14—H14	120.1
Br1—Au2—Br1^i^	173.60 (2)	C15—C14—H14	120.1
Br1—Au2—Au1	93.201 (11)	C14—C15—C16	119.9 (4)
Br1^i^—Au2—Au1	93.201 (11)	C14—C15—H15	120.1
Au2—Br1—Br1^ii^	126.21 (2)	C16—C15—H15	120.1
C11—P1—C21	107.45 (15)	C15—C16—C11	120.2 (3)
C11—P1—C1	106.44 (16)	C15—C16—H16	119.9
C21—P1—C1	108.84 (15)	C11—C16—H16	119.9
C11—P1—S1	109.38 (12)	C22—C21—C26	119.9 (3)
C21—P1—S1	113.24 (12)	C22—C21—P1	120.2 (3)
C1—P1—S1	111.20 (12)	C26—C21—P1	119.7 (3)
P1—S1—Au1	98.34 (4)	C21—C22—C23	119.4 (4)
C1^iii^—C1—P1	111.1 (3)	C21—C22—H22	120.3
C1^iii^—C1—H1A	109.4	C23—C22—H22	120.3
P1—C1—H1A	109.4	C24—C23—C22	120.5 (4)
C1^iii^—C1—H1B	109.4	C24—C23—H23	119.8
P1—C1—H1B	109.4	C22—C23—H23	119.8
H1A—C1—H1B	108.0	C25—C24—C23	119.8 (4)
C16—C11—C12	119.5 (3)	C25—C24—H24	120.1
C16—C11—P1	118.5 (3)	C23—C24—H24	120.1
C12—C11—P1	121.8 (3)	C24—C25—C26	120.8 (4)
C13—C12—C11	119.6 (4)	C24—C25—H25	119.6
C13—C12—H12	120.2	C26—C25—H25	119.6
C11—C12—H12	120.2	C25—C26—C21	119.5 (4)
C12—C13—C14	121.0 (4)	C25—C26—H26	120.2
C12—C13—H13	119.5	C21—C26—H26	120.2
			
S1—Au1—Au2—Br1	−65.75 (3)	C11—C12—C13—C14	−0.2 (6)
S1^i^—Au1—Au2—Br1	114.25 (2)	C12—C13—C14—C15	0.2 (6)
S1—Au1—Au2—Br1^i^	114.24 (3)	C13—C14—C15—C16	0.1 (6)
S1^i^—Au1—Au2—Br1^i^	−65.75 (3)	C14—C15—C16—C11	−0.4 (6)
Au1—Au2—Br1—Br1^ii^	158.03 (3)	C12—C11—C16—C15	0.5 (5)
C11—P1—S1—Au1	−172.78 (12)	P1—C11—C16—C15	176.2 (3)
C21—P1—S1—Au1	67.42 (12)	C11—P1—C21—C22	−98.2 (3)
C1—P1—S1—Au1	−55.51 (12)	C1—P1—C21—C22	146.9 (3)
Au2—Au1—S1—P1	156.28 (4)	S1—P1—C21—C22	22.7 (3)
C11—P1—C1—C1^iii^	67.1 (4)	C11—P1—C21—C26	76.0 (3)
C21—P1—C1—C1^iii^	−177.3 (3)	C1—P1—C21—C26	−38.9 (3)
S1—P1—C1—C1^iii^	−51.9 (4)	S1—P1—C21—C26	−163.1 (3)
C21—P1—C11—C16	−70.7 (3)	C26—C21—C22—C23	1.1 (6)
C1—P1—C11—C16	45.7 (3)	P1—C21—C22—C23	175.3 (3)
S1—P1—C11—C16	166.0 (2)	C21—C22—C23—C24	−0.6 (6)
C21—P1—C11—C12	104.9 (3)	C22—C23—C24—C25	−0.3 (6)
C1—P1—C11—C12	−138.7 (3)	C23—C24—C25—C26	0.7 (6)
S1—P1—C11—C12	−18.4 (3)	C24—C25—C26—C21	−0.2 (6)
C16—C11—C12—C13	−0.2 (5)	C22—C21—C26—C25	−0.7 (6)
P1—C11—C12—C13	−175.7 (3)	P1—C21—C26—C25	−174.9 (3)

Symmetry codes: (i) −*x*+1, *y*, −*z*+1/2; (ii) −*x*+1, −*y*+2, −*z*+1; (iii) −*x*+1, −*y*+1, −*z*+1.

### Hydrogen-bond geometry (Å, º)

**Table:**

*D*—H···*A*	*D*—H	H···*A*	*D*···*A*	*D*—H···*A*
C16—H16···Br1^iii^	0.95	2.81	3.712 (4)	159
C26—H26···Br1^iii^	0.95	2.89	3.775 (4)	155

Symmetry code: (iii) −*x*+1, −*y*+1, −*z*+1.
